# Supplementary material for: MetaQTL: a package of new computational methods for the meta-analysis of QTL mapping experiments
Source: BMC Bioinformatics. 2007 Feb 8;8:49. doi: 10.1186/1471-2105-8-49 (PMC1808479; doi:10.1186/1471-2105-8-49)
Supplement: Additional File 7 — MetaQTL Package : jar file and tutorial. This Zip archive contains both the MetaQTL JAR file and the files of the tutorial. [file 1471-2105-8-49-S7.zip › org.inra.metaqtl/doc/org/metaqtl/main/CmdLineParser.UnknownOptionException.html]

CmdLineParser.UnknownOptionException


|  |  |  |
| --- | --- | --- |
| **Overview** \| **Package** \| **Class** \| **Use** \| **Tree** \| **Deprecated** \| **Index** \| **Help** | |  |
| **PREV CLASS**   **NEXT CLASS** | **FRAMES**    **NO FRAMES**     **All Classes** |  |
| SUMMARY: NESTED \| FIELD \| CONSTR \| METHOD | DETAIL: FIELD \| CONSTR \| METHOD |  |


---


## org.metaqtl.main Class CmdLineParser.UnknownOptionException

```
java.lang.Object
  java.lang.Throwable
      java.lang.Exception
          org.metaqtl.main.CmdLineParser.OptionException
              org.metaqtl.main.CmdLineParser.UnknownOptionException
```

**All Implemented Interfaces:**: java.io.Serializable

**Direct Known Subclasses:**: CmdLineParser.NotFlagException, CmdLineParser.UnknownSuboptionException

**Enclosing class:**: CmdLineParser

---

``` public static class CmdLineParser.UnknownOptionException extends CmdLineParser.OptionException ```

Thrown when the parsed command-line contains an option that is not
recognised. `getMessage()` returns
an error string suitable for reporting the error to the user (in
English).

**See Also:**: Serialized Form

---

**Constructor Summary**

`CmdLineParser.UnknownOptionException(java.lang.String optionName)`

`CmdLineParser.UnknownOptionException(java.lang.String optionName,
java.lang.String msg)`


| **Method Summary** | |
| --- | --- |
| `java.lang.String` | `getOptionName()` |

**Methods inherited from class java.lang.Throwable**

`fillInStackTrace, getCause, getLocalizedMessage, getMessage, getStackTrace, initCause, printStackTrace, printStackTrace, printStackTrace, setStackTrace, toString`

**Methods inherited from class java.lang.Object**

`clone, equals, finalize, getClass, hashCode, notify, notifyAll, wait, wait, wait`

**Constructor Detail**

### CmdLineParser.UnknownOptionException

```
CmdLineParser.UnknownOptionException(java.lang.String optionName)
```

---


### CmdLineParser.UnknownOptionException

```
CmdLineParser.UnknownOptionException(java.lang.String optionName,
                                     java.lang.String msg)
```


**Method Detail**

### getOptionName

```
public java.lang.String getOptionName()
```

:   **Returns:**: the name of the option that was unknown (e.g. "-u")


---


|  |  |  |
| --- | --- | --- |
| **Overview** \| **Package** \| **Class** \| **Use** \| **Tree** \| **Deprecated** \| **Index** \| **Help** | |  |
| **PREV CLASS**   **NEXT CLASS** | **FRAMES**    **NO FRAMES**     **All Classes** |  |
| SUMMARY: NESTED \| FIELD \| CONSTR \| METHOD | DETAIL: FIELD \| CONSTR \| METHOD |  |


---
